# A high-throughput 96-well plate method to measure human uterine contractility

**DOI:** 10.3389/fphar.2026.1953934

**Published:** 2026-10-12

**Authors:** Emilie Huon, Justine Rayssac, Marie-Charlotte Lamau, Carole Abo, Gauthier Dietrich, Ylann Abrahami, Jordan Kouby, Julien Adam, Nicolas R. Chevalier

**Affiliations:** 1 Laboratoire Matières et Systèmes Complexes, Université Paris Cité, CNRS, Paris, France; 2 Service de Chirurgie Gynécologique, Hôpital Paris Saint-Joseph, Hôpital Marie Lannelongue, Paris, France; 3 Service d’Anatomie pathologique, Hôpitaux Paris Saint-Joseph Marie-Lannelongue, Paris, France

**Keywords:** adenomyosis, dyscontractility, dysmenorrhea, high-throughput screening, ion channels, muscle relaxant, non-pregnant myometrium, uterine smooth muscle

## Abstract

Uterine smooth muscle dysfunction is a common factor in many gynecological conditions, such as endometriosis, adenomyosis, dysmenorrhea, infertility, and labor dystocia. Here, we present a novel high-throughput, optical, low-cost, 96-well plate-based method for measuring *ex-vivo* uterine contractility. We validate this new method on non-pregnant human myometrial samples obtained from hysterectomies by systematically comparing contraction frequency, amplitude, and duration with the standard isometric force transducer approach. We illustrate the potential of this method by screening 8 different compounds, including hormones and pharmacological agents. In particular, we found that antagonists of T-type calcium channels (Z944) and chloride channels (benzbromarone) both decreased contractility in the non-pregnant human uterus. This method will accelerate the pharmacological development of myometrium-specific relaxing agents and provide a powerful tool to investigate the dysregulation of myogenic contractility in gynecological pathologies.

## 1 Introduction

The myometrium is the thickest smooth muscle of the human body: ∼2.5 cm in the non-pregnant female. Its menstrual-cycle-dependent peristaltic contractions (De Ziegler et al., 2001) play a major role in propelling sperm up the uterine cavity at ovulation (Kunz et al., 1996) and in evacuating menses (Lyons et al., 1991). Uterine dysmotility is associated with many gynecological pathologies. Hyper-contractility has been established in endometriosis (Bulletti et al., 2002), where it likely plays an etiogenic role by favoring retrograde transport of menstrual debris in the abdominal cavity (Sampson, 1927; Kunz et al., 2005; Vercellini et al., 2024). Adenomyosis is characterized by a thickened uterine junctional zone (Novellas et al., 2012) and poor contraction coordination (Rees et al., 2024). High uterine contraction rates are correlated with low *in-vitro* fertilization success rates (Masroor et al., 2023). Preterm contractions in pregnancy are frequent, with an estimated ∼10% pre-term birth rate worldwide (Bradley et al., 2025): preterm birth complications remain the number one cause of under-5 child mortality.

In spite of the central role played by the myometrium in women’s health, the mechanisms governing uterine smooth muscle (USM) contraction (Wray et al., 2015) remain poorly understood compared to its vascular and intestinal counterparts. Effective relaxation treatments for endometriosis, adenomyosis or infertility would ideally target specifically USM, with only minor side-effects on vascular, intestinal or airway SM. The development of such substances requires a thorough understanding of USM pathway specificities, and of their dependence on menstrual cycle phase. Several promising candidates, like atosiban (Simsek et al., 2012; Arrowsmith et al., 2016), histone deacetylase inhibitors valproic acid and trichostatin A (Moynihan et al., 2008; Liu et al., 2010) or sildenafil (Dmitrovic et al., 2013) are under investigation. The standard USM contractility assay involves tying up USM strips obtained from cesarian section (pregnancy term) or hysterectomy (non-pregnant) to transducers and measuring the tone and phasic contractions in an organ bath. Standard multi-organ bath setups present with 2–8 transducers in parallel (Crankshaw et al., 2014; Arrowsmith et al., 2018) to increase experimental output. Given the intrinsically low rhythm of uterine contractions (∼2 per 10 min), establishing a complete drug dose-dependence curve for only one sample with replica can take up to a day.

This low-throughput is a barrier to investigating the mechanisms and patient-specific profiles of myometrium contractility, which can depend on a broad range of parameters: tissue source (cesarian vs. hysterectomy), age, underlying gynecological condition, potential prior hormonal treatment, menstrual cycle phase, and exposure to non-hormonal medical treatment. Increasing the yield of contractility experiments would allow for faster screening of uterine-active compounds, identification of pathology-related dyscontractility, and the development of targeted therapies for uterine contractility disorders.

In an effort to increase the throughput of USM contractility experiments and aid drug development, we developed a novel approach based on *optical* monitoring of millimeter-sized fragments of human myometrium distributed in 96 well-plates. Our protocol offers a 10–20x throughput increase compared to the force transducer setup. This approach was inspired by a report presenting a similar methodology on non-rhythmically contracting rat aorta rings (Borges et al., 2016). We present the essential steps to set up this assay, show that rhythmic contractility can be observed in 91% of the wells, validate that this new method yields comparable results to the classical force transducer approach, and finally demonstrate its potential by examining the effect of several USM relaxing agents.

## 2 Materials and equipment

### 2.1 Study design and patient cohort

Human myometrial biopsies (n = 31) were obtained from women who underwent hysterectomy at Hôpital Paris Saint-Joseph (France). Patient cohort information is presented in Table 1. Hysterectomy was performed in the majority of cases for adenomyosis, leiomyoma, or the two combined. Ethical approval from the GERM (Groupe Ethique Recherche Médicale) was granted under IRB number 00012157. All women were provided with written information about the study and consented to participate.

**TABLE 1:** Patient cohort description.

Variables	Patients n = 31
Age (median) [IQR]	46 [43–49]
Parity (median) [IQR]	1 [1–2]
Hormonal treatment (%) [N]	19 [6]
Menstrual cycle % [N]	
— Unknown	52 [16]
— Menses	13 [4]
— Proliferative phase	16 [5]
— Secretory phase	19 [6]
Menopause (%) [N]	6 [2]
Pathology % [N]	
— Adenomyosis	71 [22]
— Fibroids	26 [8]
— Hyperplasia	3 [1]
Secondary pathology (%) [N]	
— Adenomyosis + endometriosis	10 [3]
— Adenomyosis + fibroids	39 [12]
— Hyperplasia + fibroids	3 [1]

Clinical characteristics of the n = 31 patients who underwent hysterectomy and from whom myometrial biopsies were obtained. Quantitative variables (age, parity) are expressed as median [interquartile range]; qualitative variables (hormonal treatment, menstrual cycle phase, menopausal status, pathology) are expressed as percentage [number of patients]. Menstrual cycle phase was not documented for 16 patients. Secondary pathology refers to a second condition diagnosed alongside the primary indication for hysterectomy.

### 2.2 Tissue collection and handling

After surgery, the uterus was opened by sectioning the anterior wall along the cervix-to-fundus axis. A ∼30 × 10 × 10 mm block of myometrium was cut out from the anterior wall, midway between the cervix and the fundus. The block spanned the full thickness of the uterus wall from serosa to endometrium (longest dimension of the explant, ∼30 mm). When myomas were present, myometrium was collected from regions that were devoid of them. The block was transported fresh to the laboratory in ice-cold, pre-oxygenated (95% O_2_, 5% CO_2_) medium (DMEM:F-12 + GlutaMAX, Gibco, Thermo Fisher). Tissue handling times were: hysterectomy to sample excision 15 min, transport to the lab 45 min, preparation of slices (see below) and 96 well-plate 1 h. Myometrium fragments were then incubated in oxygenated medium at 37 °C for ∼2 h before the first motility videos were recorded. The total time between hysterectomy and the first recording was thus ∼4 h.

### 2.3 Tissue sectioning and well plate preparation

The block was first cut into strips (10–20 × 2 × 1 mm), using a Feather trimming knife handle fitted with a Feather carbon-steel trimming blade (130 mm; ref. FEAT205500000, Feather, Japan). Myometrial strips were embedded in low-melting-point agarose gel (3% w/v) (Agarose LM, Condalab, Spain). The agarose block was glued to a support plate with surgical adhesive (Vetbond, 3M). The plate was mounted on a McIlwain tissue chopper (Cavey Laboratory Engineering Co. Ltd., distributed by Phymep, Paris, France), serial 1 mm-thick slices were cut, and any remaining agarose was removed. A single myometrial slice was placed inside each well of a 96-well U-bottom plate prefilled with 350 µL of medium pre-oxygenated for 10 min with carbogen (95% O_2_, 5% CO_2_) and equilibrated to 37 °C. Two gel blocks, each containing four to five tissue strips, were generally sufficient to fill one plate.

### 2.4 Drug preparation

Compounds were dissolved in the most appropriate vehicle (distilled water, 100% ethanol, or DMSO) and dispensed into a V-bottom 96-well plate according to the plate layout. The plate was sealed with clear polyethylene sealing tape to prevent evaporation before delivery into the U-bottom 96-well plate. Compound stock concentrations, vehicles, and references are provided in Supplementary Table S1.

### 2.5 Video acquisition

Explants were maintained in a custom-made chamber at 37 °C in DMEM:F-12 in a carbogen atmosphere (95% O_2_, 5% CO_2_) until phasic contractions appeared, typically within 1–3 h. Temperature was maintained at 37 °C using a transparent ITO (indium tin oxide)-coated heating plate (Thermo Plate, model TPX/Type TL Bas, Tokai Hit, Japan) mounted on a heated stage insert (22 × 17 cm, ref. 11533269, Leica Microsystems, Germany). Time-lapse videos were then acquired at 0.5 frames per second (fps) with a Basler 5472 × 3648 pixel camera (ref. V020ACA547217UMMED) fitted with a 25-mm C-mount lens, using Pylon Viewer software (v6.2.0; Basler AG, Germany). The high resolution of the camera ensured a sufficient resolution of ∼400 × 400 pixel per well. The well-plate was uniformly illuminated with a LED plate located above the chamber. Drugs were then delivered with a P10 multichannel micropipette. Motility was recorded before administration for 20 min and after administration for 30 min. Contractility parameters were quantified before drug administration and in time window 10–30 min after administration.

### 2.6 Image analysis and contractility quantification

The video processing workflow is illustrated in Supplementary Figure S1. Videos were first treated in Fiji/ImageJ. The plate was first cropped out, and any lateral drift occurring during acquisition was corrected with the Linear Stack Alignment with SIFT plugin. Well backgrounds were removed before segmentation by applying a custom macro (cercle_ROI.ijm; available at https://github.com/emiliehuon/uterus_analysis_contractility) that automatically generated 96 circular ROIs centered on the wells. Images were binarized using the Default thresholding method. Explant area was measured over the full stack automatically for each ROI using a custom Analyze-Particles macro (AnalyseParticles.ijm). The macro returned, for each ROI, the segmented explant area in every frame of the stack.

The resulting area time-series were analyzed with a MATLAB peak detection script (peak_detection.m) to extract three contractility parameters: contraction frequency (contractions per 10 min), amplitude, and contraction duration (peak width at half-prominence, in minutes). Non-contracting fragments (9%) were considered non-viable and excluded from the analysis.

The area time series of each well was first normalized to the maximal area reached by that sample during recording, which corresponds to its most relaxed state, and the contractility expressed as a dimensionless deformation:

$$Deformation\;\left(t\right)\;\left(\%\right)=\left(\frac{A_{\text{relax}}-A\left(t\right)}{A_{\text{relax}}\;}\right)100$$

where A_relax_ is the maximal (most relaxed) area reached during the recording, and A(t) the explant area at time t. The normalized traces were then smoothed with a Gaussian filter. Contraction frequency is the number of detected peaks, expressed per 10 min. Contraction amplitude is the average peak-to-valley height of the peaks measured and contraction duration is the average peak width at half-prominence, in minutes.

### 2.7 Force transducer organ bath

Two myometrium strips (10–20 × 5 × 1 mm) were mounted in an organ bath system (Emka Technologies, France). Baths were filled with DMEM:F-12 medium, continuously bubbled with carbogen (95% O_2_, 5% CO_2_) and maintained at 37 °C. One end of each strip was attached to a fixed hook and the other to a force transducer, and an initial tension of approximately 10 g was applied. Phasic contractions developed within 1–3 h, and we made sure they were regular for at least 30 min before hormone administration. Increasing concentrations of estrogen and progesterone were then added cumulatively every 30 min. Signals were acquired with IOX software (v.2.10.8.38, Emka Technologies, France), and the raw force values were exported and processed in MATLAB. Force traces were smoothed with a Gaussian filter (50-s window) and corrected for slow baseline drift by subtracting a moving average (750-s window). Contraction peaks were detected using a prominence threshold, and peak amplitude, frequency and duration (width at half-prominence) were quantified for each 30-min time window.

### 2.8 Statistics

Tissue was obtained from 31 different patients that we term “biological replicates”, although there was considerable heterogeneity in patient characteristics (Table 1). For each patient, 96 fragments were obtained, and non-contractile ones (9%) were excluded. In the 96-well setup, each drug condition at a given concentration was technically replicated 2–4 times, i.e., 2–4 fragments were subject to the same drug and same concentration, and the response was averaged over these 2–4 fragments. Not all drugs were tested on all patients; we specify the number of biological replicates for each in the corresponding figure legends and Supplementary Table S1. In the force transducer setup, we had only 1 technical replicate per patient. All points shown in the graphs correspond to biological replicates.

All statistical analyses of baseline characteristics were performed using GraphPad Prism (version 11) except Bland-Altman analysis that was performed in R (version 4.4.1) using the lme4 package. Age and parity (quantitative variables) are expressed as median and interquartile range (IQR), whereas hormone therapy, comorbidities, and menstrual cycle phase (qualitative variables) are reported as counts and percentages (Table 1). Normality of the data distribution was assessed using the Shapiro-Wilk test before each analysis.

For the comparison of the 96 well vs. force transducer methods we used a mixed-effects model fitted by restricted maximum likelihood (REML), with method and concentration as fixed effects and patient as a random effect; data were matched both across sub-columns (the two methods being applied to samples from the same patients) and across rows (the same samples being exposed to increasing concentrations), the model further accommodating the missing values inherent to this design. This analysis was followed by Šídák’s multiple comparisons *post hoc* test, comparing the two methods at each concentration (10 nM, 100 nM, 1 μM and 10 µM); adjusted p-values and the 95% confidence intervals (CI) of the corresponding mean differences are displayed on the graphs. For the transducer force, each data point corresponded to the raw value of an individual strip, whereas for the 96-well method each data point corresponds to the mean of four explants.

Agreement between the two acquisition methods was assessed by Bland-Altman analysis adapted for repeated measurements (Bland and Altman, 2007). Because each patient contributed one paired observation (96 well vs. force transducer) per concentration, the variance of the inter-method differences was divided into between-patient and within-patient components using a linear mixed-effects model with patient as a random effect. Limits of agreement were computed as the mean difference ± 1.96 times the square root of the total variance, and the standard error of the bias was taken from the same model. Confidence intervals for the bias and the limits of agreement were obtained by cluster bootstrap, resampling whole patients (Efron and Tibshirani, 1994). Proportional bias and homogeneity of the bias across concentrations were tested by likelihood-ratio test.

For pharmacological validation of the 96-well setup we used a one-sample t-test when the distribution was normal, or a one-sample Wilcoxon signed-rank test when normality was rejected. Results are reported as box-and-whisker plots of median, min to max, with all individual points displayed. A two-sided p-value <0.05 was considered statistically significant.

## 3 Methods

The fundamental idea of our method is to monitor contractility optically, through the rhythmic change in the area of each sample, to access data on 96 samples at once. The steps from hysterectomy to data analysis are shown in Figure 1A. The setup is photographed in Figure 1B, schematically depicted in Figure 1C. Details of the data analysis workflow are illustrated in Supplementary Figure S1. We found that the following experimental points were essential for the method to succeed:

**FIGURE 1:**
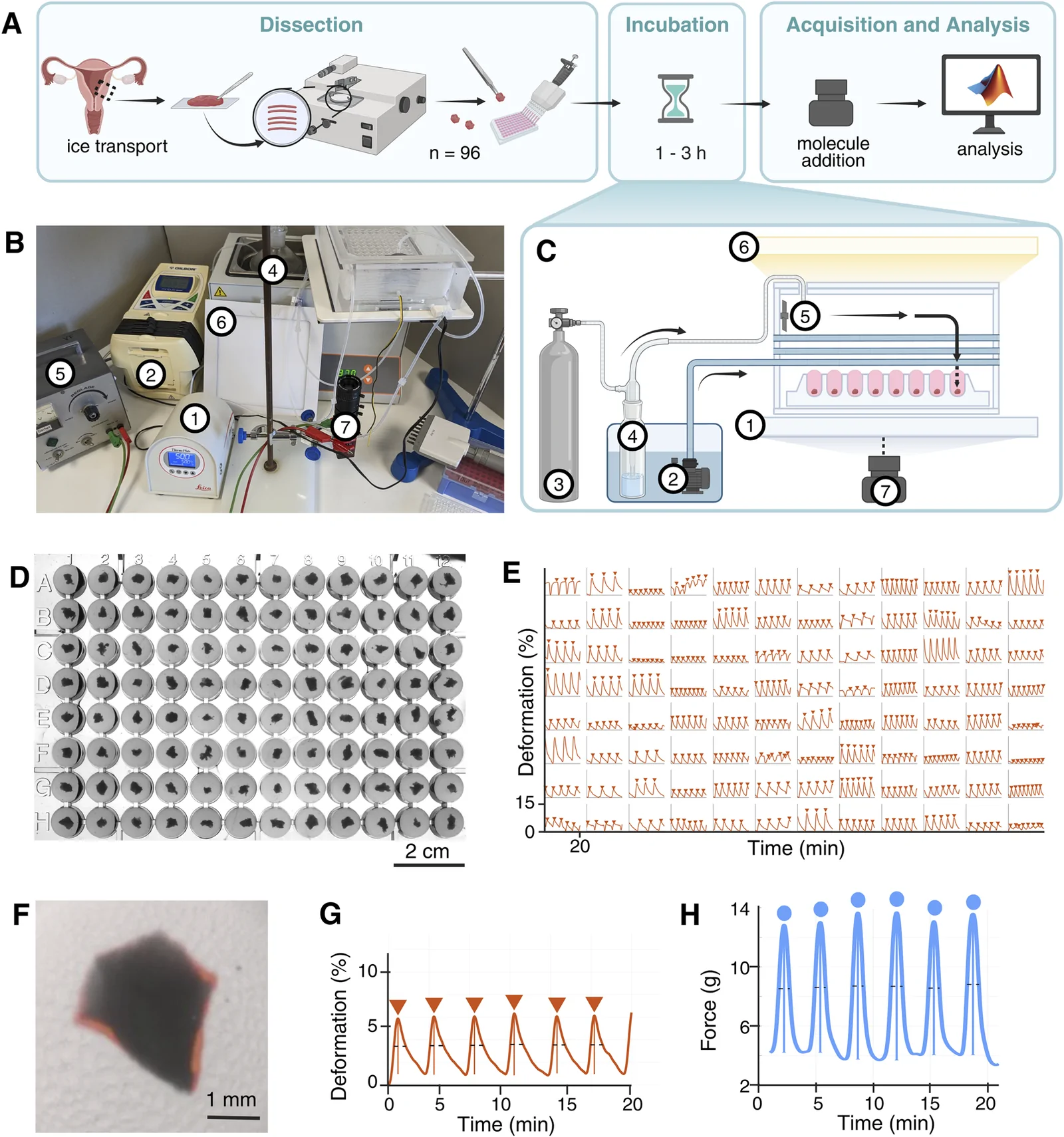
96 rhythmically beating human myometrium fragments can be recorded at once. **(A)** Experimental workflow, created with BioRender. **(B)** Photograph of the experimental setup. **(C)** Scheme of the experimental setup, created with BioRender. ① transparent ITO coated heating plate, ② circulation pump from the waterbath, ③ carbogen, ④ 37 °C Dreschel bottle, ⑤ fan, ⑥ LED panel, ⑦ HD camera. **(D)** The U-bottom 96-well plate filled with 96 myometrium fragments, size 12 × 8 cm. **(E)** Baseline contraction traces extracted from a typical experiment, showing the relative change in tissue area (%) as a function of time (min). All 96 fragments display rhythmic contractility. The 96 plots have the same scale, X-axis: 0–20 min, Y-axis: 0%–15%. **(F)** Representative area change of a fragment over one contraction cycle. The relaxed state is shown in orange, the state at contraction peak in black. **(G)** Magnification of the contraction trace extracted from a single well, showing the relative change in tissue area (%) as a function of time (min). Images were acquired at 0.5 fps. **(H)** Comparison with a representative contraction trace obtained by the classical force transducer method.

### 3.1 Tissue sectioning

The McIlwain tissue chopper is a serial “guillotine” which produces 0.1–1 mm sections from a 30 mm long strip within seconds. We found that 1 mm thick sections yielded far higher chances of eventually observing motility than 0.2 or 0.5 mm sections. This is likely due to the fact that cells in the vicinity of the section surface are dead, and that only those in the bulk of each sample can still contract. The vibratome yields better tissue surface states, but is too slow to yield 96 slices within a reasonable time.

### 3.2 U-bottom wells

Round-bottom wells ensure that each slice is centered in its well (Figure 1D) and can easily be segmented from the background; in contrast, in flat-bottom plates, slices would often stick to the well wall. The U-shape did not bring about any significant optical deformation.

### 3.3 Well replicates

The standard error of the patient-level mean decreases as the inverse square root of the number of well replicates: four wells reduce the standard error by a factor 2 compared to just one well. We used 4 replicates where plate space allowed, and 2 when several compounds were screened in parallel on the same plate (Supplementary Table S1).

### 3.4 Oxygen and heating from below

The use of carbogen (95% O_2_, 5% CO_2_) was indispensable. However, unlike in classical force transducer organ baths, we could not bubble each well. We therefore created a uniform carbogen atmosphere by injecting the gas and homogenized it inside the chamber with a small ventilator. To generate some fluid convection in each well, it is important to heat the plate from below. This was achieved with a transparent ITO-coated heating plate set to 50 °C, while the circulation at the top of the chamber was maintained at 34 °C. In doing so we ensured that the temperature at the level of each fragment was 37 °C ± 1 °C, using a wire thermometer throughout the experiment. Convection drives oxygen towards the samples and also contributes to drug mixing after administration.

### 3.5 Evaporation and condensation

The carbogen is pre-humidified and the chamber almost completely sealed to limit evaporation. Paper towels soaked with distilled water were placed around the well-plate; these are necessary to prevent condensation on the chamber lid.

### 3.6 Imaging

A high-resolution monochromatic camera (5472 × 3648 pixels) ensured a sufficient resolution of ∼400 × 400 pixels per well. A LED plate held above the chamber (not directly in contact: it heats up as well) provided uniform illumination of all wells, as long as the medium meniscus was convex (i.e., filled to the brim).

### 3.7 Drug application

Room temperature drugs, especially those dissolved in DMSO, sink to the bottom of the wells and only slowly homogenize. We therefore pre-diluted each drug in 10 times its volume of medium.

In these conditions, rhythmic phasic contractions developed spontaneously in 91% of the wells (n = 31 experiments on distinct patients) (Figure 1E, Supplementary Video S1). n = 4/31 uteri accounted for most of the non-motile fragments, these uteri having, on average, 54% non-contracting fragments. Most uteri (n = 27/31) exhibited distinct contractions in 98% of the wells (Figures 1F,G), whose frequency was comparable to that measured by the classical force transducer method (Figure 1H).

## 4 Results

### 4.1 Validation by comparison to force transducers

We systematically compared the contractility measured with this novel 96-plate methodology to the one recorded using the canonical force-transducer method, at baseline and over a log-scale range of estrogen and progesterone concentrations (Figure 2). Contraction frequency, amplitude, and duration decreased in both well-plate and force transducer assays with increasing estrogen (Figures 2A–F) and progesterone (Figures 2G–L) concentrations; both methods yielded similar dose-response curves, whether measured in absolute values (Figures 2A–C,G–I) or as percent change from the baseline of the same sample (Figures 2D–F,J–L). The baseline contraction (Figures 2B,H) cannot be directly compared with the force amplitude as they are expressed in different units, but they exhibited similar trends with increasing hormone dosage. The contraction duration (Figure 2C) was about ∼2 times longer in the 96-well protocol compared to the force-transducer method regardless of the concentration: this is likely due to the absence of sample pre-tension in the well-plate.

**FIGURE 2:**
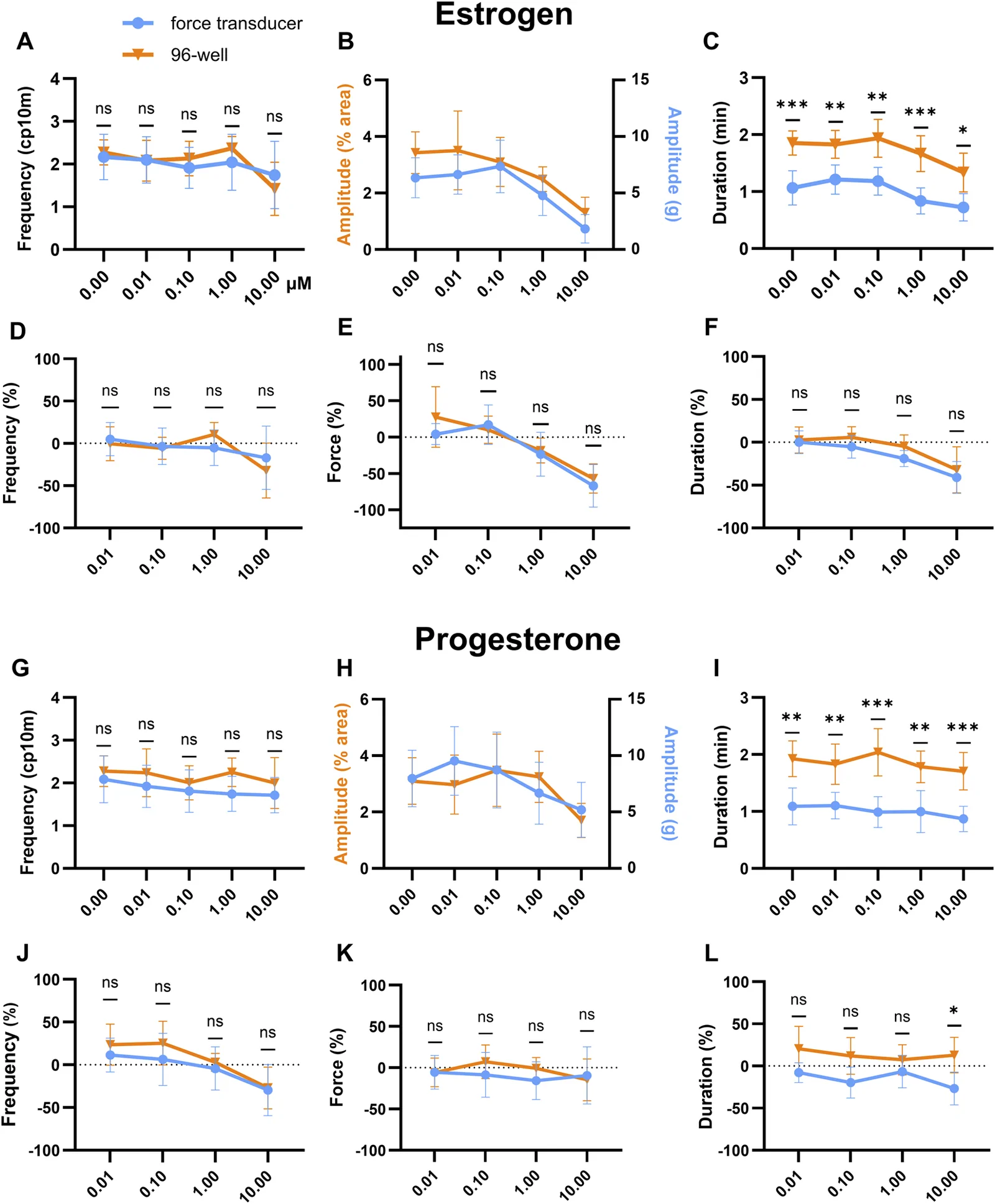
Average hormone dose-response curves measured by the novel 96-well assay and by the classical force transducer method are similar. Contraction frequency (cycles per 10 min = cp10m), amplitude, and duration in response to increasing concentrations (0, 0.01, 0.1, 1, 10 μM) of estrogen (top panel, **(A–F)**) and progesterone (bottom panel, **(G–L)**). For each hormone, the top row shows absolute values **(A–C, G–I)** while the bottom row shows the change relative to baseline (% baseline; **(D–F, J–L)**). Results from the 96-well method are shown in orange (△) and results from the force transducer method in blue (○). Data are shown as mean ± 95% CI (4 technical replicates per concentration for the 96-well method, and 1 myometrial sample per hormone for the force transducer method), obtained from n = 15 patients. Statistical comparison between acquisition methods was performed using a restricted maximum likelihood (REML). Pairwise comparisons between methods at each concentration were performed using Šídák’s multiple comparisons test. Significance of the method effect at each concentration is indicated above each pair: ns, not significant; *p < 0.05; **p < 0.01; ***p < 0.001.

### 4.2 Quantitative agreement between the 96-well and the force transducer methods

Beyond the similarity of the dose-response profiles, we quantified agreement between the two platforms using Bland-Altman analysis adapted for repeated measurements (Figure 3). Contraction frequency showed a systematic difference close to zero (Figures 3A,D). Contraction duration showed a systematic offset of 0.8–1 min (Figures 3C,F), consistent with the two-fold difference previously mentioned (Figures 2C,I). Contraction amplitude cannot be compared in absolute terms, since the optical method reports a relative area deformation (%) and the organ bath a force (g); agreement was therefore assessed on values normalized to each sample’s own pre-treatment baseline. No systematic difference was detected (bias +9.2% baseline for estrogen, +9.9% baseline for progesterone). No proportional systematic bias was detected for any parameter, and the bias was homogeneous across the concentration range.

**FIGURE 3:**
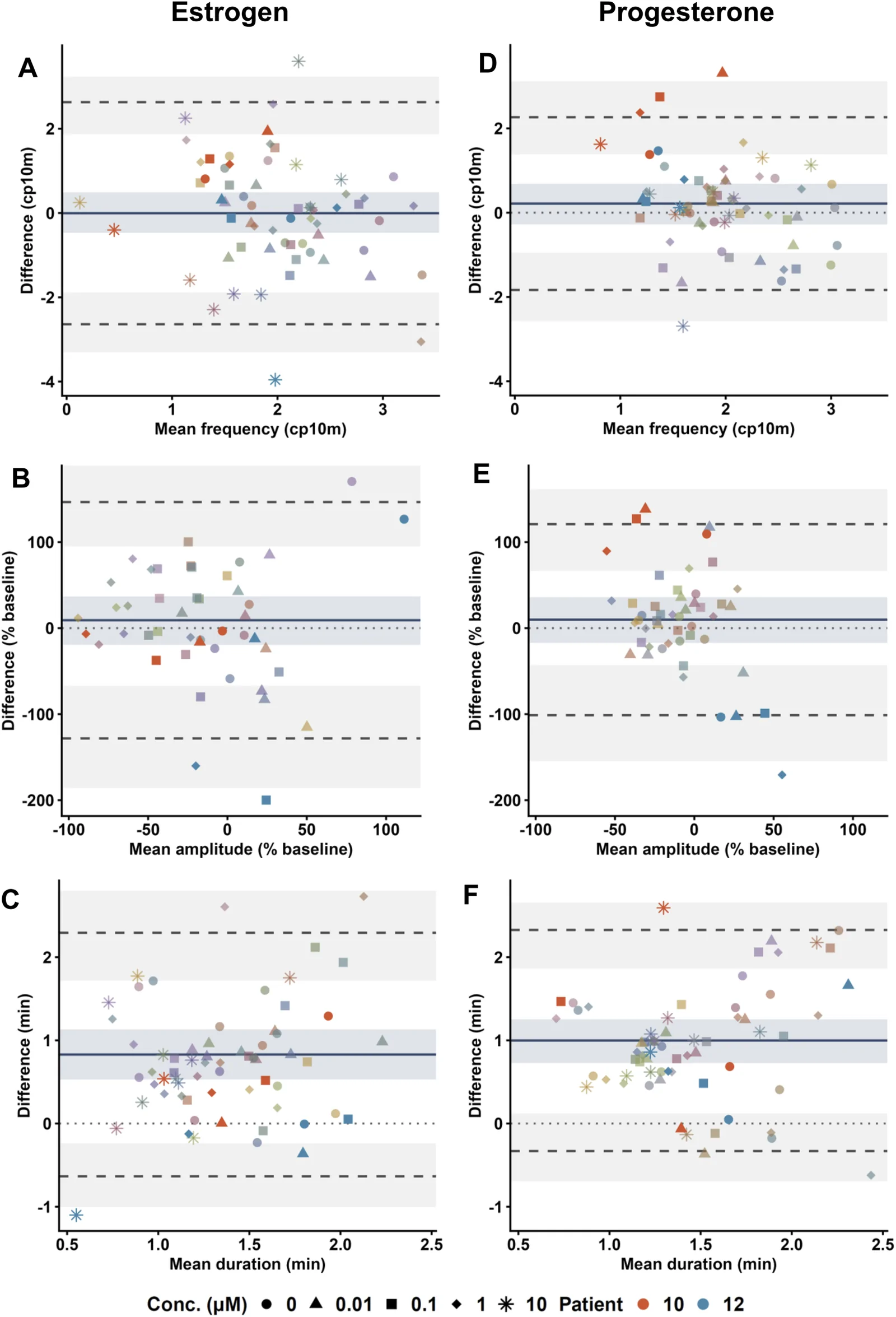
Quantitative agreement between the 96-well and the force transducer methods. Bland-Altman plots comparing the two methods for contraction frequency **(A,D)**, amplitude **(B,E)**, and duration **(C,F)**, in response to estrogen (left) and progesterone (right). The x-axis is the mean value of the measure obtained by the 96-well plate and by the force transducer. The y-axis is the difference between the 96-well assay and the force transducer: positive values indicate a higher value in the 96-well assay than with the force transducer. Frequency and duration are shown as raw values; amplitude is expressed as change from each sample’s own pre-treatment baseline. Each point is one patient at one concentration, with symbol shape denoting concentration and color denoting patient (A: 63 pairs from n = 15 patients; **(B)** 45 from n = 15; **(C)** 63 from n = 15; **(D)** 63 from n = 14; **(E)** 48 from n = 15; **(F)** 65 from n = 15). The solid line is the mean difference, and the dashed lines are the limits of agreement, computed from a mixed-effects model with patient as a random effect to account for the several paired observations contributed by each patient. Shaded bands are 95% confidence intervals: blue for the mean difference, grey for each limit of agreement. Each panel shows two outlier patients in blue and orange that fell outside the limits of agreement for estrogen and progesterone, respectively.

Bland-Altman analysis revealed that although the average response was indistinguishable between the 96-well and the force transducer methodology, individual samples could respond differently when measured in one setup or the other. High systematic (i.e., for all parameters measured) dispersion between the two methods was restricted to 2/15 patients, shown in blue and orange in Figure 3. We believe that these outliers and the wide limits of agreement stem from intrinsic heterogeneity of the myometrium: even when measured with the same device, two samples from the same uterus can yield substantially different results.

### 4.3 Validation with pharmacological compounds

We further developed the applicability and potential of our new assay by investigating the response of myometrium fragments to a range of substances known to modulate USM contractions (Figure 4). We first confirmed that drug vehicles (1:350 v/v) distilled water, DMSO and ethanol did not alter contractility, except for a small effect of DMSO on contraction duration. Tetrodotoxin (TTX) did not affect contractility either, confirming that the spontaneous activity we record is myogenic in nature; neural input to the myometrium is lost after nerve severance during hysterectomy.

**FIGURE 4:**
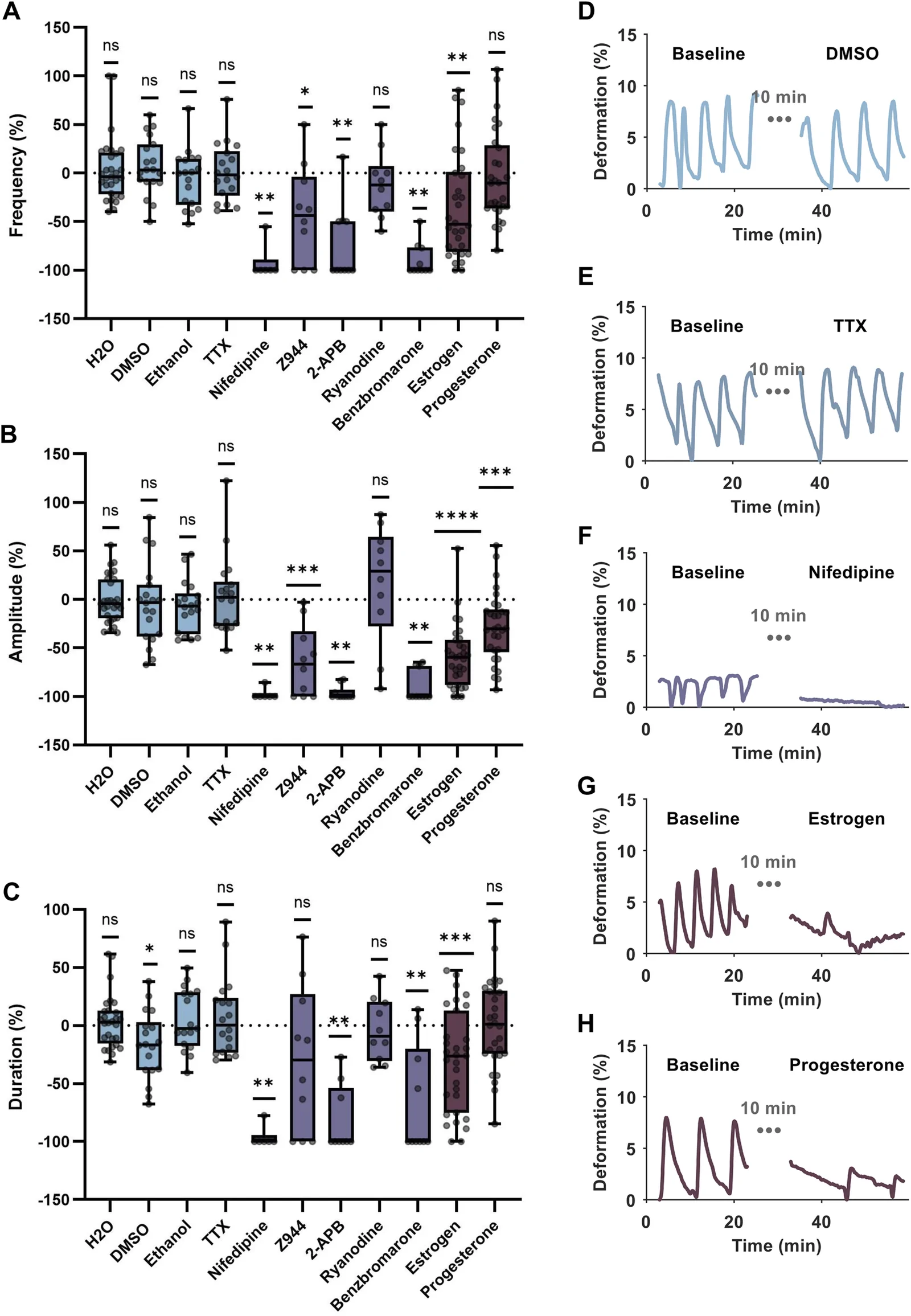
The 96-well method allows rapid screening of multiple pharmacological compounds. Effect of vehicles, drugs, and sex hormones on **(A)** contraction frequency, **(B)** amplitude, and **(C)** duration expressed as percentage change from baseline (% baseline). The effect of a drug on a given fragment was compared to its own pre-administration baseline. Each point represents one patient and is the mean of 2–4 technical replicates (2–4 wells, *cf*. Supplementary Table S1). n values listed below correspond to the number of biological replicates (distinct patients). From left to right: drug vehicles (distilled water 1:350 n = 29, DMSO 1:350 n = 18, ethanol 1:350 n = 18); the voltage-gated sodium channel blocker tetrodotoxin (TTX 1 μM, n = 18); the L-type Ca^2+^ channel antagonist nifedipine (3 μM, n = 6); the T-type Ca^2+^ channel antagonist Z944 (10 μM, n = 10); the IP_3_ receptor inhibitor 2-APB (100 μM, n = 10); the ryanodine receptor blocker ryanodine (10 μM, n = 10); the ANO1/ANO2 chloride channel blocker benzbromarone (100 μM, n = 10); and the sex hormones estrogen (10 μM, n = 30) and progesterone (10 μM, n = 29). Box-and-whisker plots show the median, interquartile range, and minimum-to-maximum. Statistical significance was assessed against the baseline using a one-sample t-test, or a one-sample Wilcoxon signed-rank test when data were not normally distributed; normality was tested using the Shapiro-Wilk test. ns, not significant; *p < 0.05; **p < 0.01; ***p < 0.001; ****p < 0.0001. **(D–H)** Representative raw contractility traces. Each trace shows change in tissue area (%) relative to the relaxed state over time for a single well, recorded before and after compound administration; the interruption marks the 10-min interval between the two acquisitions, during which the chamber was opened for drug delivery. Traces in **(D–F)** are from one patient (DMSO, TTX, and nifedipine, tested on the same plate), and traces in **(G,H)** are from another patient (estrogen and progesterone, tested on the same plate).

We next tested the dependence of contractility on Ca^2+^ channels. The L-type Ca^2+^ channel antagonist nifedipine, used as a tocolytic in the management of preterm labour (Hossain et al., 2025), abolished contractions at 0.3, 3 and 30 µM (Supplementary Figure S2). USM is also known to express Ca_V_3.1 and Ca_V_3.3 T-type Ca^2+^ channels. We found that Z944, a T-type calcium channel antagonist in clinical development for the treatment of essential tremor, significantly reduced frequency and amplitude, although to a lesser extent than nifedipine. Since we only tested Z944 at one concentration (10 µM), we cannot however formally exclude an off-target effect on Ca_v_1.2 (IC_50_ 32 µM).

Smooth muscle contractility involves binding of inositol 1,4,5-trisphosphate (IP_3_) to IP_3_ receptors (IP_3_R) on the endoplasmic reticulum downstream of cytoplasmic Ca^2+^ channel opening, triggering the release of Ca^2+^ from intracellular stores, a necessary amplification mechanism. Consistent with these presumed roles, we found that 2-APB, an inhibitor of IP_3_R, completely abolished contractility in 6/10 patients and had an inhibitory effect in 4/10 patients. We however note that 2-APB at 100 µM blocks many other cell components, including store-operated Ca^2+^ channels, gap junction connexins, potassium channels, volume-regulated anion channels (Chokshi et al., 2012); more specific IP3R inhibition could be achieved with xestospongin and by performing a dose-response assay. Ryanodine receptors (RyR) fulfil a similar Ca^2+^ amplification role in skeletal and cardiac muscle, but their contribution to smooth muscle (Guerrero-Hernández et al., 2002) and, in particular, USM (Wray and Arrowsmith, 2021) contractility is minor compared to IP_3_R (Guerrero-Hernández et al., 2002). We confirmed here that ryanodine did not alter any of the 3 contractility parameters.

The Ca^2+^-dependent chloride channel ANO1 and ANO2 blocker benzbromarone was recently found to reduce contractility in experiments performed on uterine cesarian section strips (at pregnancy term) (Bernstein et al., 2014; Danielsson et al., 2018). We found that benzbromarone (100 µM) nearly abolished USM contractility also in non-pregnant myometrium. We however caution that benzbromarone could also interact with other chloride channels besides ANO1 and ANO2.

We finally present the patient-by-patient results obtained with the sex hormones previously considered, estrogen and progesterone, at the highest concentration (10 µM), and for a higher number of patients (not all were compared with the force transducer as presented in Figures 2, 3). Estrogen and Progesterone both reduced USM contractility (Figure 4), consistent with our previous results.

## 5 Discussion

We presented a new method to optically and simultaneously record the spontaneous contractility and inotropic effects of 96 myometrial fragments. This represents a throughput increase by a factor 10 to 20 compared to classical force transducer organ bath setups. We first presented the key experimental steps required to reproduce the well setup in other laboratories. We validated that contraction frequency measured by this new method is identical to that measured by the classical force-transducer approach, and found that the two methods yield nearly the same dose-dependence curves in response to estrogen and progesterone. We finally examined a range of drug vehicles, pharmacological agents and sex hormones with known effects on USM contractility, and confirmed their expected mechanism of action using the new 96-well plate methodology.

The contraction frequencies measured by both methods at baseline were identical: ∼2 per 10 min at baseline, consistent with other investigators (Arrowsmith et al., 2012). Differences in contraction duration observed between the two acquisition methods can be attributed to the mechanical configuration of the preparations. In the force-transducer setup, the amplitude measured is a force (N) and the myometrial strip is submitted to an initial stretch which pulls back the sample after it has contracted: this results in shorter contraction durations compared to free-floating fragments (Figures 2, 3).

Bland-Altman analysis revealed that the inter-patient averaged baseline contractility and response to progesterone and estrogen are identical, but that results can differ substantially between the two methods for a given biological sample. We believe this to be due: 1°) sample heterogeneity, i.e., even two in appearance identical samples from the same patient measured by the same method can exhibit different responses (which is why technical replication is important), 2°) intrinsic sample differences between the 96-well and force-transducer methods: the fragments from the well method are much smaller, taken from different locations of the myometrium along the endometrium-to-serosa axis, and can therefore display different estrogen and progesterone receptor expressions compared to the bigger tissues that were hooked to the force transducer. The wide limits of agreement mean the optical method cannot predict the force-transducer value of an individual replicate. At the group level, however, both methods measured the same concentration responses.

Beyond throughput, the plate format changes the experimental design itself. In an organ bath, concentrations are added cumulatively to the same strip, so each response is measured after exposure to all lower concentrations. In the 96-well protocol, every concentration is applied to a separate explant at the same time and for the same duration. Responses at high concentrations are therefore not underestimated by receptor desensitization or by prior exposure, which matters for compounds such as oxytocin (Balki et al., 2013) or acetylcholine (Ehlert et al., 2001).

The complete abolition of contractions by nifedipine, down to 0.3 µM, confirms that excitation–contraction coupling in the non-pregnant human myometrium depends on calcium influx through L-type Ca_v_1.2 channels. This sensitive dependence provides a rationale for the use of nifedipine as a relaxant outside of pregnancy. A therapeutic window where nifedipine inhibits uterine motility with only minor side effects on blood pressure and intestinal transit could represent a promising route.

The T-type Ca^2+^ channel blocker Z944 significantly reduced contraction amplitude, but had a more moderate effect on frequency, and no significant effect on contraction duration. This may indicate that T-type channels are not involved in sustaining the plateau phase of the contraction. Ca_v_3 channels therefore appear as modulators of the initiation and intensity of contractions rather than of their maintenance. This may also define a therapeutic window in dysmenorrhoea: attenuating contractility without abolishing uterine activity altogether, in contrast to L-type Ca^2+^ blockade. Z944 targets all three T-type calcium channels (Ca_V_3.1, Ca_V_3.2, Ca_V_3.3), with a 60–500 fold specificity over Ca_v_1.2 (IC_50_ 32 µM). Since we applied Z944 at 10 μM, we cannot formally exclude off-target effects on Ca_v_1.2, but our result corroborates the contraction frequency decrease in human myometrium previously reported with T-type channel nickel blockade (Blanks et al., 2007).

Our data further confirm the intracellular calcium signalling pathway controlling uterine contractility. The results obtained with 2-APB and ryanodine indicate that calcium release from the endoplasmic reticulum of myometrial smooth muscle cells occurs through IP_3_ receptors rather than RyR (Figure 4). We note that 2-APB at 100 µM blocks many other cell components, including store-operated Ca^2+^ channels, sarcoplasmic-endoplasmic reticulum Ca^2+^-ATPase (SERCA) pumps, gap junction connexins, potassium channels, volume-regulated anion channels (Chokshi et al., 2012). While we cannot formally attribute the observed effect to the sole inhibition of IP_3_R, our result is consistent with previous reports placing the Gq/PLC/IP3 pathway as a point of pharmacological convergence downstream of multiple receptors (Molnar and Hertelendy, 1995; Arrowsmith and Wray, 2014; Hossain et al., 2022). The absence of reaction to ryanodine is consistent with the low expression of RyR reported in the human myometrium relative to IP_3_R, and with the absence of functional calcium-induced calcium release in this tissue (Kupittayanant et al., 2002; Noble et al., 2009; Wray and Arrowsmith, 2021).

The near-abolition of contractions by benzbromarone extends to the non-pregnant uterus previous results obtained on cesarian section myometrium (Bernstein et al., 2014; Danielsson et al., 2018). Because of the inverted chloride concentration gradient in smooth muscle cells, Ca^2+^-activated chloride channels induce a depolarization that further opens voltage-gated Ca_v_1.2, constituting a positive-feedback loop (Wray and Taggart, 2024). ANO1 and ANO2 are both expressed in human myometrium (Danielsson et al., 2018). The mechanism of action of benzbromarone should however be interpreted with caution. While this compound is known to inhibit Ca^2+^-activated ANO1 and ANO2 chloride channels (Huang et al., 2012), it is unclear whether it does not inhibit other chloride channels as well. It could in this respect behave alike broad chloride channel inhibitors NPPB or niflumic acid. From a translational standpoint, benzbromarone was introduced in the 1970s for the treatment of gout and recently withdrawn from numerous markets following cases of idiosyncratic hepatotoxicity. It remains, however, widely used in Asia; a reassessment of this drug (Lee et al., 2008) and of the necessary doses to relax the uterus seems a relevant therapeutic avenue for uterine dyscontractility.

Estrogen decreased contraction in this study, a result that may appear counterintuitive given its classically pro-contractile role mediated by increased oxytocin receptor expression (Richter et al., 2004). This apparent contradiction is resolved by the distinction between genomic and non-genomic effects: within minutes, and at high concentrations, estrogen exerts a rapid relaxant effect through activation of BK-Ca and K-ATP channels, direct inhibition of L-type calcium channels, and GPER/NO-cGMP signalling (Vedernikov et al., 2003; Tica et al., 2015). The timescale of our protocol, 10–30 min after application, excludes any transcriptional contribution and therefore places our observations within the non-genomic register.

Progesterone reduced amplitude contraction, in agreement with its physiological role in maintaining uterine quiescence and with its clinical use in the prevention of preterm birth. Here again, the rapid kinetics confirm a membrane-initiated mechanism (Knock et al., 2001; Haoui et al., 2025) in addition to a well-described transcriptional regulation effect (Tan et al., 2012; Nadeem et al., 2016).

We stress that tissue response to both estrogen and progesterone may depend sensitively on patient age (Arrowsmith et al., 2012), underlying gynecological condition, potential prior hormonal treatment, menstrual cycle phase, and exposure to non-hormonal medical treatment. We were deliberately broad in our inclusion criteria for this study because the main aim of the study was to compare the 96-well vs. force transducer for a given tissue, irrespective of its biological characteristics. In spite of these many confounding factors, the average tissue responses we observed using either method reproduced results observed previously by other investigators (Knock et al., 2001; Vedernikov et al., 2003; Tica et al., 2015).

Some limitations of our study should be noted. First, we observed that the first 5–10 min after drug administration (after opening and closing the chamber) cannot generally be exploited because of the transient cooling of the medium; careful preparation of the drug plate beforehand minimizes this dead time. Second, concentration–response relationships were only established for estrogen and progesterone, and not for most other compounds.

The pharmacological data generated with this optical acquisition method demonstrate the power of the approach for identifying drugs targeting uterine hypercontractility. By measuring the respective contributions of L- and T-type calcium channels, chloride channels and IP_3_-mediated sarcoplasmic reticulum calcium release, this work validates this new high-throughput protocol for further development of myometrium-selective uterine relaxants, and for the investigation of the fundamental mechanisms of uterine contractility. The 96-well format is well suited for the establishment of dose-response curves and the determination of IC50/EC50. It also has the potential of screening patient specific factors in dyscontractility, allowing both a comparison of baseline contractility across patients with different pathological backgrounds, and of their differential response to drugs and hormones.

## Data Availability

The raw data supporting the conclusions of this article will be made available by the authors, without undue reservation.

## References

[B1] Arrowsmith S. Wray S. (2014). Oxytocin: its mechanism of action and receptor signalling in the myometrium. J. Neuroendocrinol. 26, 356–369. 10.1111/JNE.12154 24888645

[B2] Arrowsmith S. Robinson H. Noble K. Wray S. (2012). What do we know about what happens to myometrial function as women age? J. Muscle Res. Cell Motil. 33, 209–217. 10.1007/s10974-012-9300-2 22644420 PMC3413813

[B3] Arrowsmith S. Neilson J. Wray S. (2016). The combination tocolytic effect of magnesium sulfate and an oxytocin receptor antagonist in myometrium from singleton and twin pregnancies. Am. J. Obstet. Gynecol. 215, 789.e1–789.e9. 10.1016/j.ajog.2016.08.015 27555315

[B4] Arrowsmith S. Keov P. Muttenthaler M. Gruber C. W. (2018). Contractility measurements of human uterine smooth muscle to aid drug development. J. Vis. Exp. 131, e56639. 10.3791/56639 29443077 PMC5841565

[B5] Balki M. Erik-Soussi M. Kingdom J. Carvalho J. C. A. (2013). Oxytocin pretreatment attenuates oxytocin-induced contractions in human myometrium *in vitro* . Anesthesiology 119, 552–561. 10.1097/ALN.0B013E318297D347 23676375

[B6] Bernstein K. Vink J. Y. Fu X. W. Wakita H. Danielsson J. Wapner R. (2014). Calcium-activated chloride channels anoctamin 1 and 2 promote murine uterine smooth muscle contractility. Am. J. Obstet. Gynecol. 211, 688.e1–688.e10. 10.1016/j.ajog.2014.06.018 24928056 PMC4253652

[B7] Bland J. M. Altman D. G. (2007). Agreement between methods of measurement with multiple observations per individual. J. Biopharm. Stat. 17, 571–582. 10.1080/10543400701329422 17613642

[B8] Blanks A. M. Zhao Z. H. Shmygol A. Bru-mercier G. Astle S. Thornton S. (2007). Characterization of the molecular and electrophysiological properties of the T-type calcium channel in human myometrium. J. Physiol. 581, 915–926. 10.1113/JPHYSIOL.2007.132126 17446221 PMC1976399

[B9] Borges R. Díaz-Martín D. Hernández-Jiménez J. G. Rodríguez-Valido M. Beltrán B. (2016). Analyzing isolated blood vessel contraction in multi-well plates. Naunyn. Schmiedeb. Arch. Pharmacol. 389, 521–528. 10.1007/s00210-016-1218-6 26905519

[B10] Bradley E. Blencowe H. Moller A. B. Okwaraji Y. B. Sadler F. Gruending A. (2025). Born too soon: global epidemiology of preterm birth and drivers for change. Reprod. Health 22, 105. 10.1186/S12978-025-02033-X 40551192 PMC12186353

[B11] Bulletti C. Ziegler D. D. Polli V. Del Ferro E. Palini S. Flamigni C. (2002). Characteristics of uterine contractility during menses in women with mild to moderate endometriosis. Fertil. Steril. 77, 1156–1161. 10.1016/S0015-0282(02)03087-X 12057721

[B12] Chokshi R. Fruasaha P. Kozak J. A. (2012). 2-Aminoethyl diphenyl borinate (2-APB) inhibits TRPM7 channels through an intracellular acidification mechanism. Channels 6, 362–369. 10.4161/CHAN.21628 22922232 PMC3508775

[B13] Crankshaw D. J. Sweeney E. M. Obrien Y. M. Walsh J. M. Dockery P. Morrison J. J. (2014). The influence of smooth muscle content and orientation in dissected human pregnant myometrial strips on contractility measurements. Eur. J. Pharmacol. 738, 245–249. 10.1016/j.ejphar.2014.05.037 24886879

[B14] Danielsson J. Vink J. Hyuga S. Fu X. W. Funayama H. Wapner R. (2018). Anoctamin channels in human myometrium: a novel target for tocolysis. Reprod. Sci. 25, 1589–1600. 10.1177/1933719118757683 29471754 PMC6344949

[B15] De Ziegler D. Bulletti C. Fanchin R. Epiney M. Brioschi P. A. (2001). Contractility of the nonpregnant uterus: the follicular phase. Ann. N. Y. Acad. Sci. 943, 172–184. 10.1111/j.1749-6632.2001.tb03801.x 11594539

[B16] Dmitrovic R. Kunselman A. R. Legro R. S. (2013). Sildenafil citrate in the treatment of pain in primary dysmenorrhea: a randomized controlled trial. Hum. Reprod. 28, 2958–2965. 10.1093/HUMREP/DET324 23925396 PMC3795469

[B17] Efron B. Tibshirani R. J. (1994). An Introduction to the Bootstrap. Chapman and Hall/CRC. 10.1201/9780429246593

[B18] Ehlert F. J. Ansari K. Z. Shehnaz D. Sawyer G. W. Griffin M. T. (2001). Acetylcholine-induced desensitization of muscarinic contractile response in guinea pig ileum is inhibited by pertussis toxin treatment. J. Pharmacol. Exp. Ther. 299, 1126–1132. 10.1016/s0022-3565(24)29237-5 11714903

[B19] Guerrero-Hernández A. Gómez-Viquez L. Guerrero-Serna G. Rueda A. (2002). Ryanodine receptors in smooth muscle. Front. Biosci. 7, d1676–d1688. 10.2741/A871 12086921

[B20] Haoui M. Vergara C. Kenzler L. Schröer J. Zimmer-Bensch G. Fleck D. (2025). Kir7.1 is the physiological target for hormones and steroids that regulate uteroplacental function. Sci. Adv. 11, eadr5086. 10.1126/SCIADV.ADR5086 40043131 PMC11881918

[B21] Hossain M. R. Tolosa J. M. Young R. C. Smith R. Paul J. W. (2022). Assessing the potency of the novel tocolytics 2-APB, Glycyl-H-1152, and HC-067047 in pregnant human myometrium. Reprod. Sci. 30 (1), 203–220. 10.1007/S43032-022-01000-2 35715551 PMC9810572

[B22] Hossain M. R. Paul M. Tolosa J. M. Smith R. Paul J. W. (2025). Preventing preterm birth: the search for tocolytic synergism. Reprod. Sci. 32 (10), 3262–3286. 10.1007/S43032-025-01941-4 40855143 PMC12546381

[B23] Huang F. Zhang H. Wu M. Yang H. Kudo M. Peters C. J. (2012). Calcium-activated chloride channel TMEM16A modulates mucin secretion and airway smooth muscle contraction. Proc. Natl. Acad. Sci. U. S. A. 109, 16354–16359. 10.1073/PNAS.1214596109 22988107 PMC3479591

[B24] Knock G. A. Tribe R. M. Hassoni A. A. Aaronson P. I. (2001). Modulation of potassium current characteristics in human myometrial smooth muscle by 17β-Estradiol and progesterone. Biol. Reprod. 64, 1526–1534. 10.1095/BIOLREPROD64.5.1526 11319161

[B25] Kunz G. Beil D. Deininger H. Wildt L. Leyendecker G. (1996). The dynamics of rapid sperm transport through the female genital tract: evidence from vaginal sonography of uterine peristalsis and hysterosalpingoscintigraphy. Hum. Reprod. 11, 627–632. 10.1093/HUMREP/11.3.627 8671281

[B26] Kunz G. Beil D. Huppert P. Noe M. Kissler S. Leyendecker G. (2005). Adenomyosis in endometriosis--prevalence and impact on fertility. Evidence from magnetic resonance imaging. Hum. Reprod. 20, 2309–2316. 10.1093/HUMREP/DEI021 15919780

[B27] Kupittayanant S. Luckas M. J. M. Wray S. (2002). Effect of inhibiting the sarcoplasmic reticulum on spontaneous and oxytocin-induced contractions of human myometrium. BJOG 109, 289–296. 10.1111/J.1471-0528.2002.01110.X 11950184

[B28] Lee M. H. H. Graham G. G. Williams K. M. Day R. O. (2008). A benefit-risk assessment of benzbromarone in the treatment of gout. Was its withdrawal from the market in the best interest of patients? Drug Saf. 31, 643–665. 10.2165/00002018-200831080-00002 18636784

[B29] Liu X. Yuan L. Guo S. W. (2010). Valproic acid as a therapy for adenomyosis: a comparative case series. Reprod. Sci. 17, 904–912. 10.1177/1933719110373807 20601534

[B30] Lyons E. A. Taylor P. J. Xin H. Z. Ballard G. Levi C. S. Kredentser J. V. (1991). Characterization of subendometrial myometrial contractions throughout the menstrual cycle in normal fertile women. Fertil. Steril. 55, 771–774. 10.1016/S0015-0282(16)54246-0 2010002

[B31] Masroor M. J. Asl L. Y. Sarchami N. (2023). The effect of uterine contractions on fertility outcomes in frozen embryo transfer cycles: a cohort study. J. Reprod. Infertil. 24, 132. 10.18502/jri.v24i2.12498 37547572 PMC10402453

[B32] Molnar M. Hertelendy F. (1995). Signal transduction in rat myometrial cells: comparison of the actions of endothelin-1, oxytocin and prostaglandin F2α. Eur. J. Endocrinol. 133, 467–474. 10.1530/EJE.0.1330467 7581972

[B33] Moynihan A. T. Hehir M. P. Sharkey A. M. Robson S. C. Europe-Finner G. N. Morrison J. J. (2008). Histone deacetylase inhibitors and a functional potent inhibitory effect on human uterine contractility. Am. J. Obstet. Gynecol. 199, 167.e1–167.e7. 10.1016/j.ajog.2008.01.002 18455134

[B34] Nadeem L. Shynlova O. Matysiak-Zablocki E. Mesiano S. Dong X. Lye S. (2016). Molecular evidence of functional progesterone withdrawal in human myometrium. Nat. Commun. 7, 11565. 10.1038/NCOMMS11565 27220952 PMC4894948

[B35] Noble K. Matthew A. Burdyga T. Wray S. (2009). A review of recent insights into the role of the sarcoplasmic reticulum and Ca entry in uterine smooth muscle. Eur. J. Obstetrics Gynecol. Reproductive Biol. 144, S11–S19. 10.1016/j.ejogrb.2009.02.010 19285773

[B36] Novellas S. Chassang M. Delotte J. Toullalan O. Chevallier A. Bouaziz J. (2012). MRI characteristics of the uterine junctional zone: from normal to the diagnosis of adenomyosis. AJR. Am. J. Roentgenol. 196, 1206–1213. 10.2214/AJR.10.4877 21512093

[B37] Rees C. O. Thomas S. de Boer A. Huang Y. Zizolfi B. Foreste V. (2024). Quantitative ultrasound measurement of uterine contractility in adenomyotic vs. normal uteri: a multicenter prospective study. Fertil. Steril. 121, 864–872. 10.1016/j.fertnstert.2024.01.009 38199285

[B38] Richter O. N. Kübler K. Schmolling J. Kupka J. Reinsberg J. Ulrich U. (2004). Oxytocin receptor gene expression of estrogen-stimulated human myometrium in extracorporeally perfused non-pregnant uteri. Mol. Hum. Reprod. 10, 339–346. 10.1093/MOLEHR/GAH039 15044599

[B39] Sampson J. A. (1927). Peritoneal endometriosis due to the menstrual dissemination of endometrial tissue into the peritoneal cavity. Am. J. Obstet. Gynecol. 14, 422–469. 10.1016/s0002-9378(15)30003-x

[B40] Simsek Y. Celik O. Karaer A. Gul M. Yilmaz E. Koc O. (2012). Therapeutic efficiency of atosiban, an oxytocin receptor blocking agent in the treatment of experimental endometriosis. Arch. Gynecol. Obstet. 286, 777–783. 10.1007/s00404-012-2390-7 22648446

[B41] Tan H. Yi L. Rote N. S. Hurd W. W. Mesiano S. (2012). Progesterone Receptor-A and -B have opposite effects on proinflammatory gene expression in human myometrial cells: implications for progesterone actions in human pregnancy and parturition. J. Clin. Endocrinol. Metab. 97, E719–E730. 10.1210/JC.2011-3251 22419721 PMC3339884

[B42] Tica O. S. Comanescu A. Bogdan M. Alexandru D. O. Tica A. A. Ciurea T. (2015). The estrogen-induced effects on myometrium are the result of activation of two different receptor types: GPER and ERα. Curr. Health Sci. J. 41, 209–212. 10.12865/CHSJ.41.03.03 30534424 PMC6246995

[B43] Vedernikov Y. P. Hartke J. R. De Long M. A. Saade G. R. Garfield R. E. (2003). Sex hormone effects in non-pregnant rat and human myometrium. Eur. J. Obstetrics Gynecol. Reproductive Biol. 108, 59–66. 10.1016/S0301-2115(02)00415-3 12694972

[B44] Vercellini P. Piccini M. Caprara F. Cetera G. E. Viganò P. Somigliana E. (2024). Potential anatomical determinants of retrograde menstruation: a comprehensive narrative review. Reprod. Biomed. Online 49, 1–10. 10.1016/j.rbmo.2024.104345 39137508

[B45] Wray S. Arrowsmith S. (2021). Uterine excitability and ion channels and their changes with gestation and hormonal environment. Annu. Rev. Physiol. 83, 331–357. 10.1146/ANNUREV-PHYSIOL-032420-035509 33158376

[B46] Wray S. Taggart M. J. (2024). An update on pacemaking in the myometrium. J. Physiol. 604, 3661–3671. 10.1113/JP284753 39073139 PMC13134432

[B47] Wray S. Burdyga T. Noble D. Noble K. Borysova L. Arrowsmith S. (2015). Progress in understanding electro-mechanical signalling in the myometrium. Acta Physiol. 213, 417–431. 10.1111/apha.12431 25439280

