# Supplementary material for: A high-throughput 96-well plate method to measure human uterine contractility
Source: Front Pharmacol. 2026 Oct 12;17:1953934. doi: 10.3389/fphar.2026.1953934 (PMC13696793; doi:10.3389/fphar.2026.1953934)
Supplement: Supplementary file 1 [file Supplementaryfile1.pdf]

Supplementary Material

1 Supplementary Figures and Tables

1.1 Table S1 – List of compounds used

Compounds	Solvent	n (patients)	Replicate wells	Reference	Supplier
**Tetrodotoxin**	H_2O	18	4	120055	Abcam
**Nifedipine**	DMSO	6	4	N7634	Sigma
**Z944**	DMSO	10	2	6367	Biotechne
**Benzbromarone**	DMSO	10	2	B5774	Sigma
**2APB**	DMSO	10	2	1224	Biotechne
**Ryanodine**	DMSO	10	2	1329	Biotechne
**β-Estradiol**	ethanol 100%	30	4	E8875	Sigma
**Progesterone**	ethanol 100%	29	4	P8783	Sigma

1.2 Figure S1

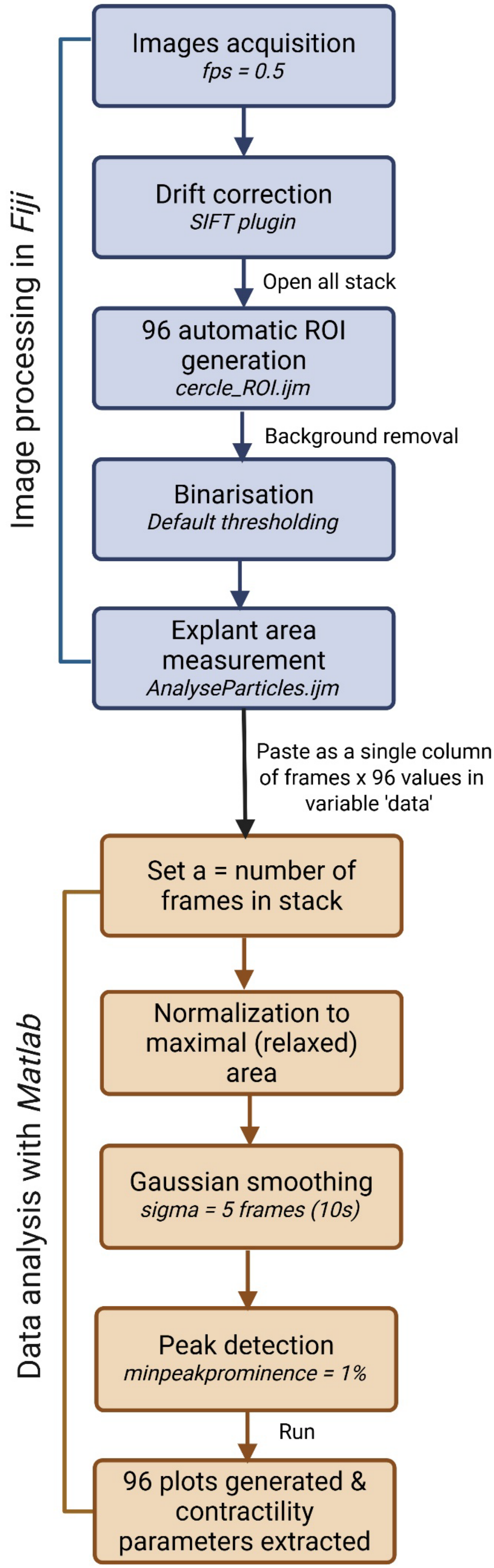
Image processing in *Fiji*
Images acquisition
fps = 0.5
Drift correction
SIFT plugin
Open all stack
96 automatic ROI generation
cercle_ROI.ijm
Background removal
Binarisation
Default thresholding
Explant area measurement
AnalyseParticles.ijm
Paste as a single column of frames x 96 values in variable 'data'
Data analysis with *Matlab*
Set a = number of frames in stack
Normalization to maximal (relaxed) area
Gaussian smoothing
sigma = 5 frames (10s)
Peak detection
minpeakprominence = 1%
Run
96 plots generated & contractility parameters extracted

Figure S1 - Workflow of image processing and data analysis.

Steps performed in Fiji (blue) and in Matlab (orange), with the parameter values used throughout the study. All scripts are available at https://github.com/emiliehuon/uterus_analysis_contractility. Created with BioRender

1.3 Figure S2

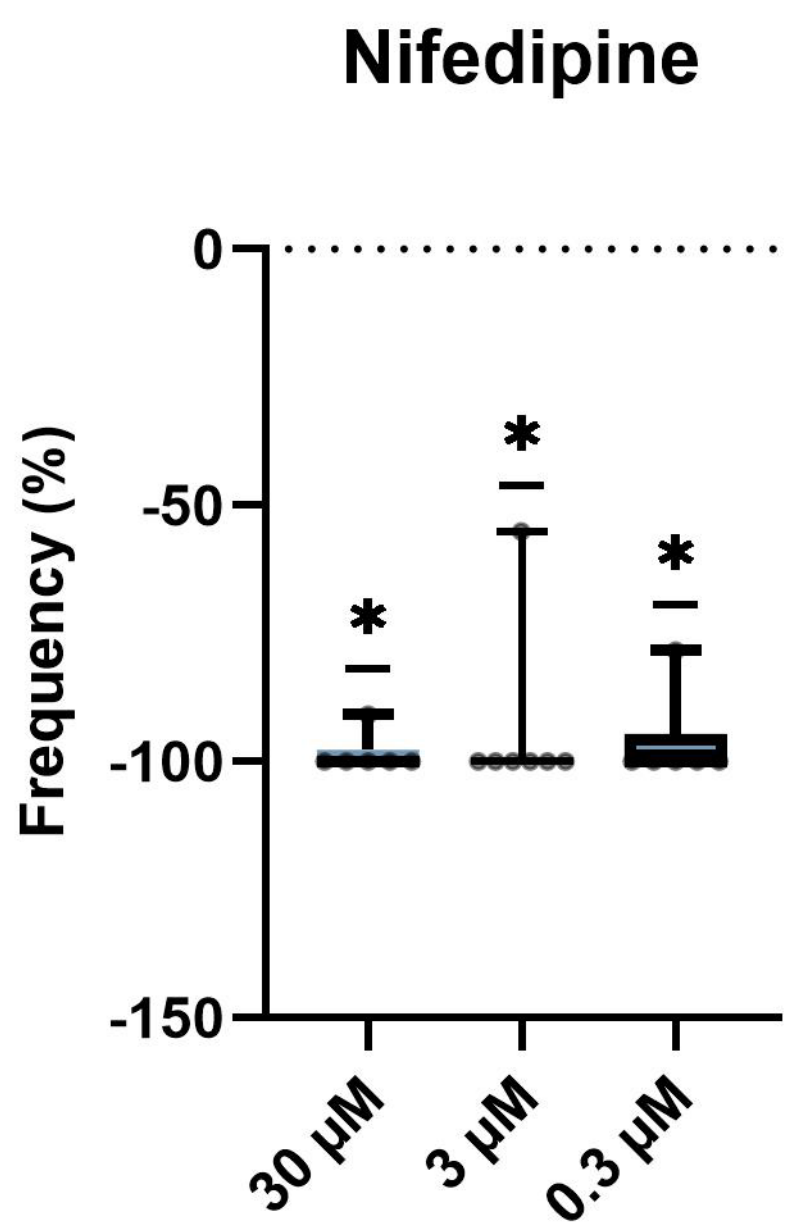

Supplementary Figure 2. Dose-response assay for nifedipine using the 96-well assay, at 0.3 µM (n=5 patients), 3 µM (n=6) and 30 µM (n=5). One-sample Wilcoxon signed-rank test, *$p<0.05$.
